# Detection and differentiation of ataxic and hypokinetic dysarthria in cerebellar ataxia and parkinsonian disorders via wave splitting and integrating neural networks

**DOI:** 10.1371/journal.pone.0268337

**Published:** 2022-06-03

**Authors:** Joomee Song, Ju Hwan Lee, Jungeun Choi, Mee Kyung Suh, Myung Jin Chung, Young Hun Kim, Jeongho Park, Seung Ho Choo, Ji Hyun Son, Dong Yeong Lee, Jong Hyeon Ahn, Jinyoung Youn, Kyung-Su Kim, Jin Whan Cho

**Affiliations:** 1 Department of Neurology and Neuroscience Center, Samsung Medical Center, Sungkyunkwan University School of Medicine, Seoul, Republic of Korea; 2 Medical AI Research Center, Research Institute for Future Medicine, Samsung Medical Center, Seoul, Republic of Korea; 3 Department of Health Sciences and Technology, SAIHST, Sungkyunkwan University, Seoul, Republic of Korea; 4 Department of Radiology, Samsung Medical Center, Sungkyunkwan University School of Medicine, Seoul, Republic of Korea; 5 Department of Data Convergence and Future Medicine, Sungkyunkwan University School of Medicine, Seoul, Republic of Korea; Politechnika Slaska, POLAND

## Abstract

Dysarthria may present during the natural course of many degenerative neurological conditions. Hypokinetic and ataxic dysarthria are common in movement disorders and represent the underlying neuropathology. We developed an artificial intelligence (AI) model to distinguish ataxic dysarthria and hypokinetic dysarthria from normal speech and differentiate ataxic and hypokinetic speech in parkinsonian diseases and cerebellar ataxia. We screened 804 perceptual speech analyses performed in the Samsung Medical Center Neurology Department between January 2017 and December 2020. The data of patients diagnosed with parkinsonian disorders or cerebellar ataxia were included. Two speech tasks (numbering from 1 to 50 and reading nine sentences) were analyzed. We adopted convolutional neural networks and developed a patch-wise wave splitting and integrating AI system for audio classification (PWSI-AI-AC) to differentiate between ataxic and hypokinetic speech. Of the 395 speech recordings for the reading task, 76, 112, and 207 were from normal, ataxic dysarthria, and hypokinetic dysarthria subjects, respectively. Of the 409 recordings of the numbering task, 82, 111, and 216 were from normal, ataxic dysarthria, and hypokinetic dysarthria subjects, respectively. The reading and numbering task recordings were classified with 5-fold cross-validation using PWSI-AI-AC as follows: hypokinetic dysarthria vs. others (area under the curve: 0.92 ± 0.01 and 0.92 ± 0.02), ataxia vs. others (0.93 ± 0.04 and 0.89 ± 0.02), hypokinetic dysarthria vs. ataxia (0.96 ± 0.02 and 0.95 ± 0.01), hypokinetic dysarthria vs. none (0.86 ± 0.03 and 0.87 ± 0.05), and ataxia vs. none (0.87 ± 0.07 and 0.87 ± 0.09), respectively. PWSI-AI-AC showed reliable performance in differentiating ataxic and hypokinetic dysarthria and effectively augmented data to classify the types even with limited training samples. The proposed fully automatic AI system outperforms neurology residents. Our model can provide effective guidelines for screening related diseases and differential diagnosis of neurodegenerative diseases.

## Introduction

Dysarthria is a major clinical sign of various neurological diseases that manifests as indiscernible speech due to the dysfunction of muscles controlled by the nervous system involved in speech production. The presentation of dysarthria is distinct, depending on each disease and the causative neurological condition [1, 2]. While various types of dysarthria may present during the natural course of degenerative neurological conditions, hypokinetic and ataxic dysarthria are important in movement disorders as they represent the underlying neuropathology and are highly prevalent [3–6]. Representative neurodegenerative diseases with progressive ataxic dysarthria include multiple systemic atrophy (MSA), sporadic or inherited cerebellar ataxia, and multiple sclerosis. In Parkinson’s disease (PD) and progressive supranuclear palsy (PSP), hypokinetic dysarthria presents as an early symptom, sometimes even before cardinal signs such as resting tremor or rigidity are apparent [3, 7].

Detecting dysarthria and distinguishing between types of dysarthria is important for diagnosing the underlying disease and evaluating its progression [1]. The detection of hypokinetic dysarthria is critical owing to the growing prevalence of neurodegenerative diseases such as Alzheimer’s disease and PD, as a result of the growing aging population around the world [8, 9]. Ataxic dysarthria is also important as it is a relatively common presentation of ataxia in pediatric and adult populations due to various etiologies [10, 11]. People with movement disorders such as parkinsonian disorders or cerebellar ataxia tend to show specific types of dysarthria (hypokinetic or ataxic). Therefore, the ability to differentiate between patients who have hypokinetic or ataxic dysarthria can help in the differential diagnosis of PD, PSP subtypes, and MSA subtypes.

However, evaluation of the type and severity of dysarthria requires significant neurological knowledge and experience and is performed by expert speech specialists or neurologists. Subsequently, several automated systems that use machine or deep learning have been developed with the aim of developing efficient tools to detect dysarthria. This approach includes automated measurement of acoustic analysis values in specific dysarthria [12], detection of disease using voice recordings [13], and assessment of severity level [14]. These methods depended on the extraction of acoustic features from speech utterances such as pitch and harmonics, shimmer, and jitter, followed by their classification using traditional machine learning methods such as Gaussian mixture model (GMM), hidden Markov model (HMM), and support vector machine (SVM). Identification of acoustic and spectral features in PD is performed using Mel-frequency cepstral coefficients (MFCC), linear prediction coefficients, and GMM, achieving an accuracy of 77.6% [15]. Wu et al. addressed acoustic features using MFCC, spherical K-means, and the pooling method to detect PD and compared the accuracy of acoustic features [16]. GMM and MFCC were used in detecting Huntington’s disease by learning acoustic and lexical features of voice recordings [17].

Some studies have reported the use of a deep learning system that learned the voice recording itself, not the acoustic feature. Lauraitis et al. adopted a bidirectional long short-term memory neural network and wavelet scattering transform with SVM classifier for detecting speech impairment in patients [18]. Kumar et al. proposed a convolutional neural network (CNN) model that learned sustained vowel sounds of patients with PD [19]. Nevertheless, these studies were limited because they only assessed a small group or one type of disease (e.g., PD, Alzheimer’s) and compared it with the results from healthy controls or one type of dysarthria. Some studies used an augmentation method to overcome their small datasets with promising results [20, 21]. However, no study has applied the augmentation method on several diseases of the same speech disturbance. Learning the specific dysarthria type is more important than merely learning a disease speech because various diseases show a mixture of dysarthria type, and when AI can differentiate speech type, it can be applied to various diseases without learning every disease speech. Few studies have detected specific dysarthria types instead of diseases; for instance, Kaya et al. used a VGG19-SVM hybrid model to detect ataxia in patients with multiple sclerosis [22, 23]. However, further improvement is required for more accurate and various disease differentiation. Therefore, there is a need for advanced modeling that can mimic assessments conducted by experienced clinicians and differentiate between dysarthria types.

We sought to develop a model to distinguish ataxic dysarthria and hypokinetic dysarthria from normal speech and differentiate ataxic and hypokinetic speech in parkinsonian diseases and cerebellar ataxia. To evaluate the performance of our model, we used a CNN suitable for analyzing Mel spectrogram as the model input. To improve the diagnostic performance, we proposed and applied a patch-wise wave splitting and integrating system, which amplifies the amount of training data and improves the diagnostic generalizability.

## Materials and methods

### Study population and definition of dysarthria

We retrospectively screened perceptual speech analyses performed in the Neurology Department of the Samsung Medical Center between January 2017 and December 2020. The analyses of patients who were diagnosed with PD, atypical parkinsonian syndrome (i.e., MSA-P, and PSP), and cerebellar ataxia (i.e., MSA-C, inherited cerebellar ataxia, and sporadic adult-onset ataxia (SAOA)) were included. The diagnosis of each patient was determined based on these criteria: PD was based on the United Kingdom Parkinson’s Disease Society Brain Bank criteria [24] using 18-F N-(3-fluoropropyl)-2β-carbon ethoxy-3β-(4-iodophenyl) nortropane positron emission tomography (FP-CIT PET). Probable MSA and probable PSP were diagnosed based on the second consensus diagnosis of MSA [25] and movement disorder society clinical diagnostic criteria for PSP [26], respectively. SAOA was diagnosed based on criteria outlined in previous SAOA studies [27, 28]. Other inherited cerebellar ataxias were diagnosed when the pathologic gene was found. Patients with concomitant or structural brain lesions, including stroke, tumors, cardiopulmonary and musculoskeletal problems, or other neurological conditions (e.g., myelopathy, known neuropathy, chronic vestibular dysfunction), which may affect speech, were excluded.

Ataxic and hypokinetic dysarthria were defined according to the universal definition [29]. Experienced clinicians in speech analysis and speech therapy (MKS, SJH), specialized in the differential diagnosis of neurogenic speech and language disorders, assessed and classified the speech recordings and confirmed the type and severity of dysarthria in each patient. They classified the speech recordings independently and were not allowed to know each other’s classification. No evidence of any dysarthria, ataxic and hypokinetic dysarthria, was identified, and hereafter, we refer to no evidence of dysarthria as “none.”

This study was approved by the Institutional Review Board (IRB) of Samsung Medical Center. No informed consent from patients was required because the study was a retrospective observational study, and no figures or videos of a recognizable patient have been included (IRB number: 2021-07-026).

### Protocol for perceptual speech analysis

Patient speech was digitally recorded using a headset microphone (Shure SM48 cardioid) positioned approximately 15 cm from the subject’s mouth at a sampling rate of 44,100 Hz, using a multi-dimensional voice program (Kay Elemetrics, Lincoln Park, NJ, USA). Every recording was executed in a soundproof room with only the patient and instructor present.

Two speech tasks were given, and at most, two trials were allowed in each task. In the number task, the patients were instructed to count from 1 to 50 as fast as possible without pausing, if possible. This protocol was referred to as the number protocol (S1 File). In the autumn task, the patients were instructed to read a specific paragraph consisting of nine sentences about autumn. The patients were asked to read the sentences at their usual rate and loudness in this task. The second protocol was referred to as the autumn protocol (S2 File).

### Audio data acquisition: Patient demographics and diagnoses

A total of 804 perceptual speech analyses were screened, and 422 patients were included. Among them, 395 participated in the autumn protocol, and 409 participated in the number protocol. In the autumn protocol, 76 cases did not show evidence of dysarthria, 112 had ataxia, and 207 had hypokinetic dysarthria. In the number protocol, 82 cases did not have evidence of dysarthria, 111 were ataxic, and 216 were hypokinetic dysarthria. The number of patients in each protocol is shown in Table 1. In addition, the demographic, specific diagnosis, and clinical characteristics of the data we received are summarized and compared in Table 2.

**Table 1:** Number of patients in the autumn and number protocols.

	Autumn	Number
**Hypokinetic**	207	216
**Ataxia**	112	111
**None**	76	82
**Total**	395	409

**Table 2:** Demographics and clinical characteristics.

	Ataxic	Hypokinetic	None
**Age (years)**	65.00 ± 8.46	68.68 ± 9.40	74.00 ± 5.77
**Sex, male**	53 (47.3)	112 (51.9)	40 (48.8)
**Degree of dysarthria (mild/moderate/severe)**	50 (44.3)/57 (51.5)/ 5 (4.2)	107 (49.3)/92 (42.6)/17 (8.0)	-

Data are presented as the mean ± standard deviation (SD) or n (%).

### Audio data pre-processing

**Fig 1:**
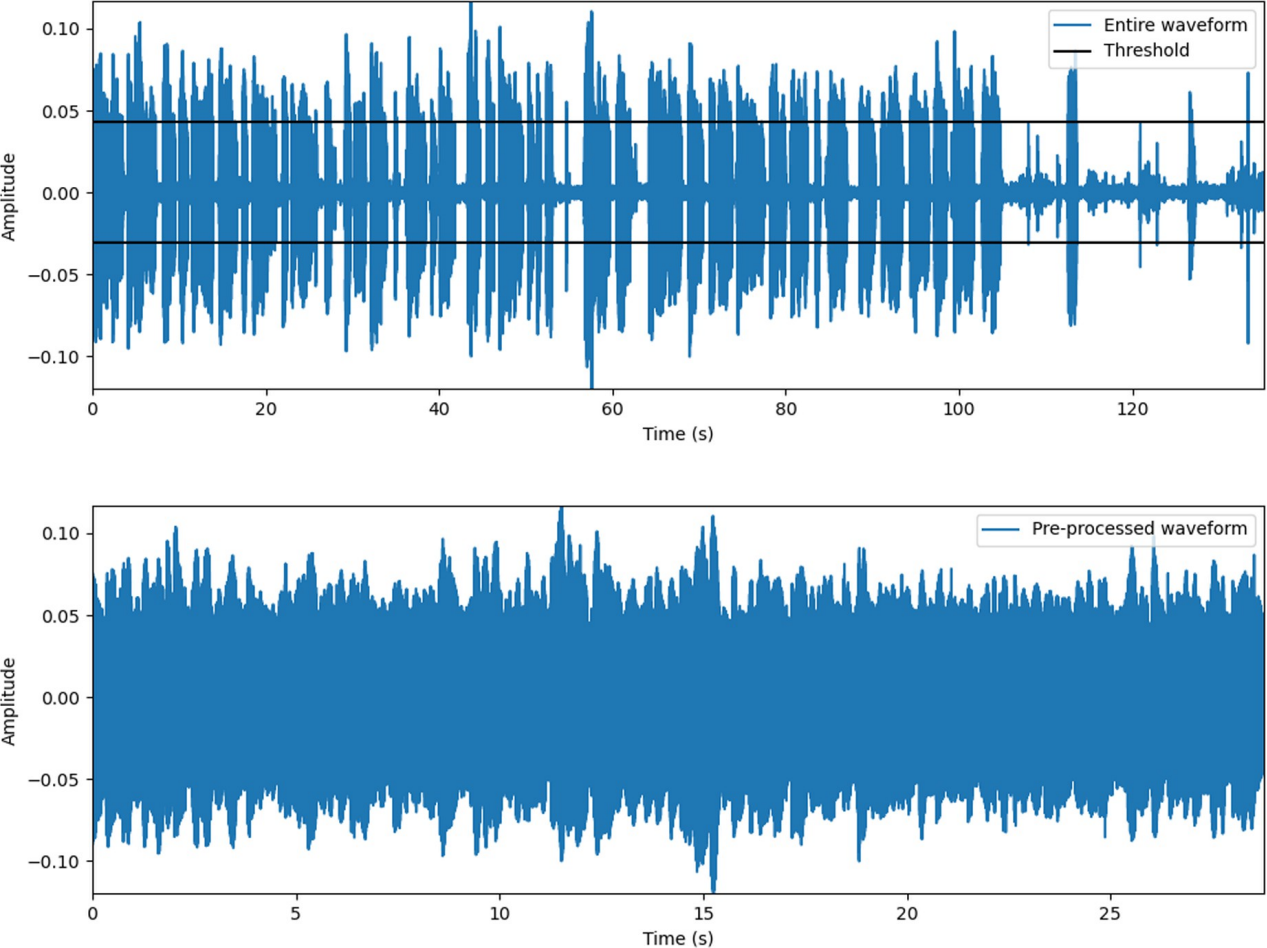
Illustration of the pre-processing process. Top: entire waveform (blue line) and threshold (black line). Bottom: pre-processed waveform where only the values above the threshold were extracted.

We performed sound source pre-processing for each of the sound sources of these two protocols so that the section from the time the patient started speaking to the time the patient finished speaking could be extracted through binary thresholding based on a specific volume level of the waveform. This audio pre-processing is illustrated in Fig 1. We obtained the average root mean square (rms) sound level of the entire waveform and defined a value corresponding to 50% of this value as the threshold. Thereafter, a waveform was newly extracted by leaving only the values of the waveform with a value greater than this threshold value. This pre-processing made it possible to effectively extract only the patient’s waveform from the entire waveform file by removing the doctor’s voice or ambient background noise.

### Algorithm overview: Patch-wise wave splitting and integrating AI system (PWSI -AI)

**Fig 2:**
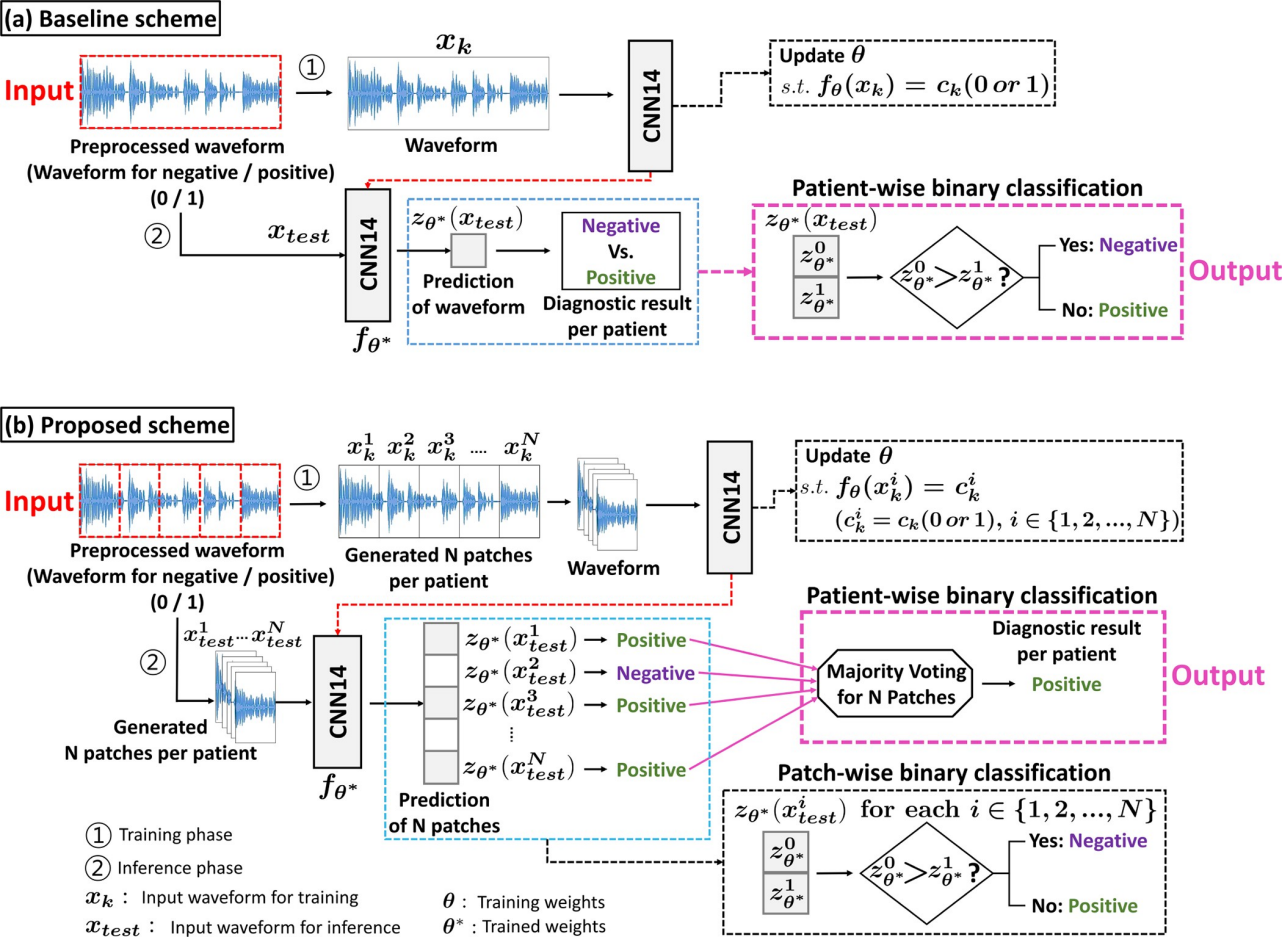
Overview of the proposed PWSI-AI-AC. (a) Baseline scheme: The network receives the entire waveform as an input and performs learning and inference for the binary classification. (b) Proposed scheme: Unlike the baseline method, the entire waveform is divided into *N* patches, and the network receives the individual patch waveform as input and learns to perform binary classification for each patch. In the inference phase, *N* results are synthesized through majority voting to obtain the final binary classification result for the target patient. Through this diversity gain, performance improvement can be achieved.

Owing to the characteristics of medical data, the number of datasets required for learning by the deep learning model is generally insufficient. We developed a patch-wise wave splitting and integrating AI system for audio classification (PWSI-AI-AC) to overcome this problem. This approach is illustrated with a comparison to the baseline model in Fig 2. To understand the characteristics of the proposed technology, we first describe the existing baseline method and then compare it to our method.

### Baseline model

An existing general AI-based model for performing speech classification receives the entire speech as the network input and learns to perform the binary classification based on the entire speech input (training phase). After completing all training, the network receives the waveform of a test patient not used for training as an input and provides a binary classification prediction result as an output (inference phase). These training and inference processes are outlined in Fig 2(A), and the approaches are considered as the general process for our baseline model. As a backbone model for the network in this study, we used CNN14 [30]. Specifically, the learning and inference processes for this baseline technique can be formulated as detailed in the following two paragraphs, respectively.

When there are training data of *D* patients, let *x*_*k*_ denote the *k*-th patient’s waveform file. The network *f*_*θ*_ then takes *x*_*k*_ as input and provides a two-dimensional probability vector *f*_*θ*_(*x*_*k*_)∈ℝ^2^ as its output as follows: For *k*∈{1,2,…,*D*},

$$f_{\theta }(x_{k})=softmax(z_{\theta }(x_{k}))=\left(\frac{e^{z_{\theta }^{0}(x_{k})}}{\sum_{i=0}^{1}e^{z_{\theta }^{i}(x_{k})}},\frac{e^{z_{\theta }^{1}(x_{k})}}{\sum_{i=0}^{1}e^{z_{\theta }^{i}(x_{k})}}\right)\in R^{2},$$ (1)

where *softmax* denotes the softmax function allowing the sum of the network outputs to be 1 (i.e., let the network output be a probability vector), *θ* denotes the network parameter for learning, and $z_{\theta }(x_{k})≔(z_{\theta }^{0}(x_{k}),\;z_{\theta }^{1}(x_{k}))\in R^{2}$ denotes the network latent feature vector before the softmax output layer.

If the *k*-th patient has a specific disease, a positive label (i.e., *c*_*k*_ = 1) is annotated; otherwise, a negative label (i.e., *c*_*k*_ = 0) is given. In this way, binary classification data for a total of *D* patients were collected as ${\left\{(x_{k},\;c_{k})\right\}}_{k=1}^{D}$. A total of five different binary classification datasets were prepared according to the type of dysarthria. Further details are introduced in the next section. Using each binary classified dataset, the network is trained to minimize the following objective:

$$\theta^{*}=\underset{\theta }{\mathrm{argmin}}\sum_{k=1}^{D}L_{bce}(c_{k},\;f_{\theta }(x_{k})),$$ (2)

where θ* indicates the trained parameter, and *L*_*bce*_ denotes the binary cross-entropy loss as specified in the following equation (l denotes the indicator function yielding 1 if the inner statement is correct):

$$L_{bce}(c_{k},\;f_{\theta }(x_{k}))=-l[c_{k}=0]\log\left(\frac{e^{z_{\theta }^{0}(x_{k})}}{\sum_{i=0}^{1}e^{z_{\theta }^{i}(x_{k})}}\right)-l[c_{k}=1]\log\left(\frac{e^{z_{\theta }^{1}(x_{k})}}{\sum_{i=0}^{1}e^{z_{\theta }^{i}(x_{k})}}\right)$$ (3)

In Eq (3), *c*_*k*_∈{0,1} is the actual label information, and the log function input value is the estimated probability value of the network for this label.

The network *f*_θ*_, trained according to Eq (2), receives the waveform *x*_*test*_ of the test patient as an input and provides an estimate of the binary classification label as its output. This step can be performed by obtaining the index of the largest value (an index having the highest probability value) among the two-dimensional probability vector values of the network as follows. For scaling, we applied log to softmax and applied the argmax function from this log-softmax value to select the corresponding index and performed binary classification:

$$\begin{array}{c} {\hat{c}}_{test}=\underset{\mathrm{index}\in \{0,1\}}{\mathrm{argmax}}\left(\log\;f_{\theta^{*}}(x_{test})\right)\\ =\underset{\mathrm{index}\in \{0,1\}}{\mathrm{argmax}}\left(\log\;softmax(z_{\theta^{*}}(x_{test}))\right)\\ =\underset{\mathrm{index}\in \{0,1\}}{\mathrm{argmax}}\left(log(\frac{e^{z_{\theta^{*}}^{0}(x_{test})}}{\sum_{i=0}^{1}e^{z_{\theta^{*}}^{i}(x_{test})}}),\;log(\frac{e^{z_{\theta^{*}}^{1}(x_{test})}}{\sum_{i=0}^{1}e^{z_{\theta^{*}}^{i}(x_{test})}})\right) \end{array}$$ (4)

Whether this label estimate ${\hat{c}}_{test}\in \{0,1\}$ is equal to the actual label *c*_*test*_∈{0,1} allows us to evaluate the binary classification performance of that baseline.

### Proposed model

The training and inference processes of the proposed PWSI-AI-AC are provided in Fig 2(B). Unlike the baseline model, the proposed PWSI-AI-AC model divides the entire speech section (*x*_*k*_) into ‘*N*’ subsections ($x_{k}^{i}$ for *i*∈{1,2,…,*N*}), assigns the same label to each subsection and trains the network to receive these individual subsections as input and output for the corresponding label (training phase). This patch-based approach augments the data ‘*N*’ times compared to the baseline, which can improve the network classification performance. After training the model, we performed the following three steps for the inference process: 1) (wave splitting) generating ‘*N*’ patches that consisted of ‘*N*’ sub-intervals for an entire waveform, 2) (generating ‘*N*’ patch-based predictions) individually inputting these ‘*N*’ patches into the trained network and obtaining a prediction value for each patch for each class as the corresponding value of the log-softmax output of the network, 3) (wave integrating) averaging the ‘*N*’ prediction values of ‘*N*’ patches for each class and deriving the final class label as the index of the largest value among these average values through the argmax operation. In other words, as the proposed method simultaneously uses *N* predictions instead of only one prediction (i.e., combining *N* predictions by a majority vote to derive the final binary classification result), unlike the existing method (i.e., baseline), it was possible to obtain improved diagnostic performance by obtaining additional gains in diversity. Furthermore, we compared how the learning and inference processes differ from the existing baseline model through the following formulations.

For the training phase of the proposed PWSI-AI-AC, given the *k*-th patient’s waveform file *x*_*k*_ from training data of *D* patients, the proposed wave splitting process generates ‘*N*’ waveform patch files (i.e., $x_{k}^{i}$ for *i*∈{1,2,…,*N*}) by dividing the corresponding waveform *x*_*k*_ into *N* independent sections in chronological order as follows:

$$x_{k}\to (x_{k}^{1},\;x_{k}^{2},\;x_{k}^{3},\ldots ,,x_{k}^{N})\;for\;k\in \{1,2,\ldots ,D\})$$ (5)

While the baseline model uses an undivided waveform *x*_*k*_ as the input, as shown in Eq (1), the proposed model takes a divided waveform patch $x_{k}^{i}$ (for *i*∈{1,2,…,*N*}) as the input and provides a two-dimensional probability vector $f_{\theta }(x_{k}^{i})\in R^{2}$ as its output as follows:

For *i*∈{1,2,…,*N*},

$$f_{\theta }(x_{k}^{i})=softmax(z_{\theta }(x_{k}^{i}))=\left(\frac{e^{z_{\theta }^{0}(x_{k}^{i})}}{\sum_{j=0}^{1}e^{z_{\theta }^{j}(x_{k}^{i})}},\frac{e^{z_{\theta }^{1}(x_{k}^{i})}}{\sum_{j=0}^{1}e^{z_{\theta }^{j}(x_{k}^{i})}}\right)\in R^{2}$$ (6)

Using a binary classified dataset as ${\left\{(x_{k},\;c_{k})\right\}}_{k=1}^{D}$, the proposed model is trained to minimize the following objective:

$$\theta^{*}=\underset{\theta }{\mathrm{argmin}}\sum_{k=1}^{D}\sum_{i=1}^{N}L_{bce}(c_{k},\;f_{\theta }(x_{k}^{i})).$$ (7)

This learning objective is similar to the baseline model but differs in that the proposed model predicts the actual label *c*_*k*_ of the waveform by using only a portion of the entire waveform as an input. As each (undivided) waveform *x*_*k*_ has a unique label, all *N* patches (i.e., $x_{k}^{i}$ for *i*∈{1,2,…,*N*}) were assigned that same label.

For the inference phase of the proposed PWSI-AI-AC, as the proposed network uses individual sections of the waveform as the input, a separate process (i.e., wave integrating) is required in the inference step to fully use the entire waveform *x*_*test*_ for the test patient. In our approach, the network *f*_θ*_, trained according to Eq (7), individually receives *N* divided waveform patches $x_{test}^{i}$ (for *i*∈{1,2,…,*N*}) of the target/original waveform *x*_*test*_ as an input and thus provides multiple *N* estimates of the binary classification label as its outputs. Each of the *N* predicted values is expressed as follows: For *i*∈{1,2,…,*N*}),

$${\hat{c}}_{test}^{i}=\underset{\mathrm{index}\in \{0,1\}}{\mathrm{argmax}}\left(\log\;f_{\theta^{*}}(x_{test}^{i})\right)=\underset{\mathrm{index}\in \{0,1\}}{\mathrm{argmax}}\left(log(\frac{e^{z_{\theta^{*}}^{0}(x_{test}^{i})}}{\sum_{j=0}^{1}e^{z_{\theta^{*}}^{j}(x_{test}^{i})}}),log(\frac{e^{z_{\theta^{*}}^{1}(x_{test}^{i})}}{\sum_{j=0}^{1}e^{z_{\theta^{*}}^{j}(x_{test}^{i})}})\right)$$ (8)

The proposed method calculates one final prediction result by synthesizing these *N* prediction values using majority voting, which is expressed as follows:

$${\hat{c}}_{test}=\underset{\mathrm{index}\in \{0,1\}}{\mathrm{argmax}}\left(\sum_{i=1}^{N}l[{\hat{c}}_{test}^{i}=0],\;\sum_{i=1}^{N}l[{\hat{c}}_{test}^{i}=1]\right)$$ (9)

Notably, this final label estimate ${\hat{c}}_{test}$ is equivalent to the result of averaging the ‘*N*’ prediction values of ‘*N*’ patches for each class and deriving the final class label as the index of the largest value among these average values through the argmax operation as follows:

$${\hat{c}}_{test}=\underset{\mathrm{index}\in \{0,1\}}{\mathrm{argmax}}\left(\frac{1}{N}\sum_{i=1}^{N}\log\;softmax(z_{\theta^{*}}(x_{test}^{i}))\right)=\underset{\mathrm{index}\in \{0,1\}}{\mathrm{argmax}}(\frac{1}{N}\sum_{i=1}^{N}log(\frac{e^{z_{\theta^{*}}^{0}(x_{test}^{i})}}{\sum_{j=0}^{1}e^{z_{\theta^{*}}^{j}(x_{test}^{i})}}),\frac{1}{N}\sum_{i=1}^{N}log(\frac{e^{z_{\theta^{*}}^{1}(x_{test}^{i})}}{\sum_{j=0}^{1}e^{z_{\theta^{*}}^{j}(x_{test}^{i})}})),$$ (10)

where the second equality is obtained by applying Eq (8). The final label prediction result obtained in Eq (10) has lower computational complexity than that in Eq (9), as it does not need to directly compute *N* individual predictions (i.e., ${\hat{c}}_{test}^{i}$ for *i*∈{1,2,…,*N*}) in Eq (8). Therefore, we calculated the final prediction as in Eq (10).

In summary, the baseline model uses only one prediction result per test patient, but the proposed model derives the diagnosis result by synthesizing *N* multiple prediction results. Therefore, as the experimental results demonstrate, our model provides higher predictive performance by exploiting the diversity gain.

### Experimental settings and implementations for AI systems

The original audio length ranged from 19 to 315 seconds (s) in the autumn protocol from 11 to 103 s in the number protocol. After all audio files were pre-processed as described in Fig 1, we resized all of these processed audio files to 30 s and use them as AI input. The baseline model used a waveform set to 30 s, adjusted in this way, as an input. In the proposed PWSI-AI-AC, the process of dividing each waveform into N was added. That is, each pre-processed sound source was divided equally into N patches and adjusted to a length of 30 s for each patch sound source in the same way as the baseline to enable a fair comparison. We in this study set the number *N* of patches as 10 and 3 for autumn and number protocols, respectively. This is because the average length of autumn protocol voice source files is approximately three times longer than that for number protocol. So we tripled the number *N* of patches as 10 in the autumn protocol compared to that for number protocol (i.e., *N* = 3) to ensure that the actual time interval covered by each patch was consistent, which showed higher performance than other configurations.

**Fig 3:**
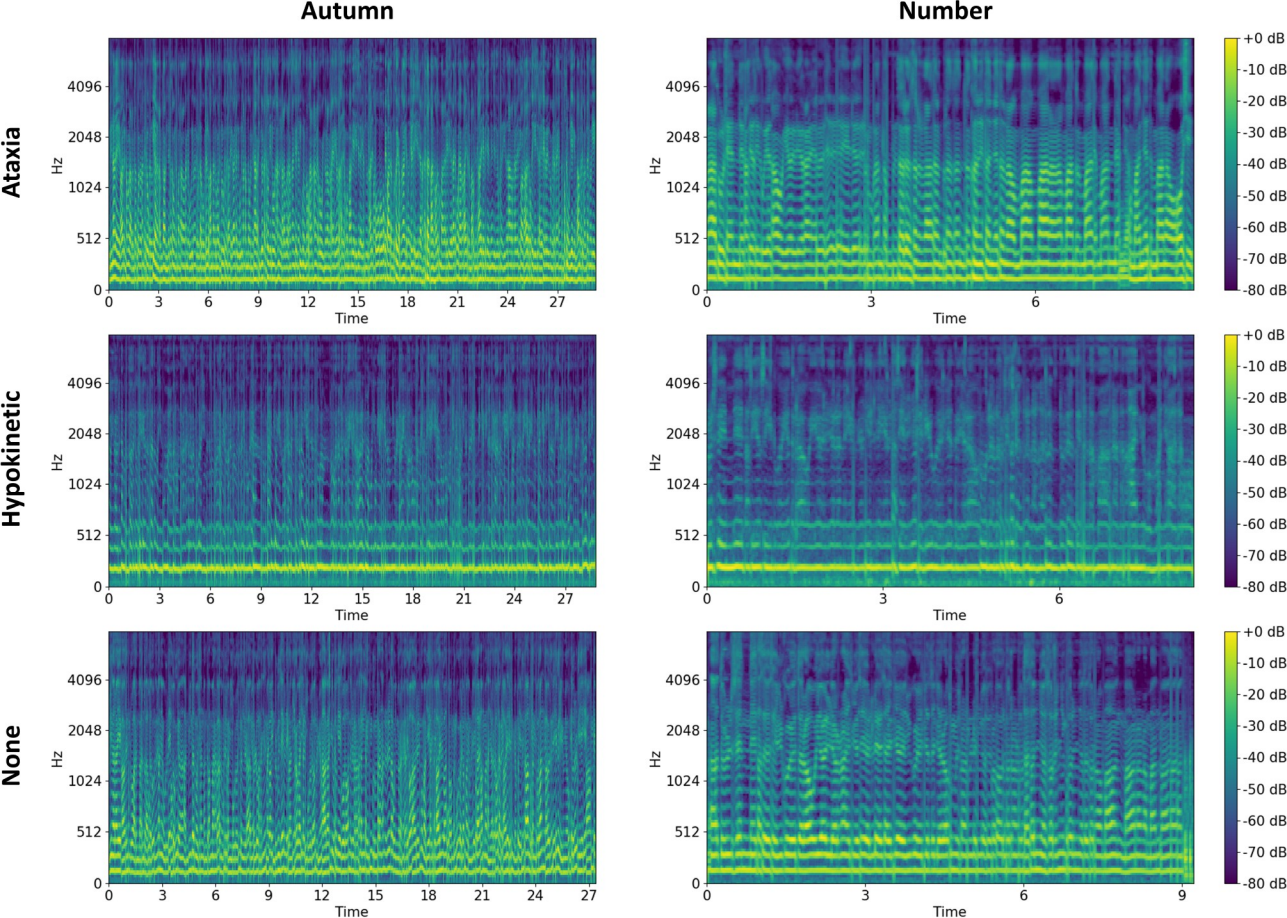
The log-Mel spectrogram of the waveform according to the selected dysarthria types (no evidence of dysarthria, hypokinetic dysarthria, and ataxic dysarthria) for each autumn and number protocol.

We applied log-Mel transformation [30, 31] to each waveform, converted it to log-Mel spectrogram, and used it as input for the AI network. Log-Mel spectrogram has already been used as an input for CNN in audio tagging to derive good performance [32, 33]. Short time Fourier transforms (STFTs) [34] are applied to time domain waveforms to calculate spectrograms. Mel filter banks are then applied to the spectrograms, followed by a logarithmic operation to extract log-Mel spectrograms [32, 33]. Therefore, a log-Mel spectrogram consisting of a time axis and frequency axis (i.e., expressing one-dimensional sound source information as two-dimensional information) can be extracted. This log-Mel spectrogram is illustrated in Fig 3 according to the selected dysarthria types for each autumn and number protocol as examples of cases where the number of patches was one.

**Fig 4:**
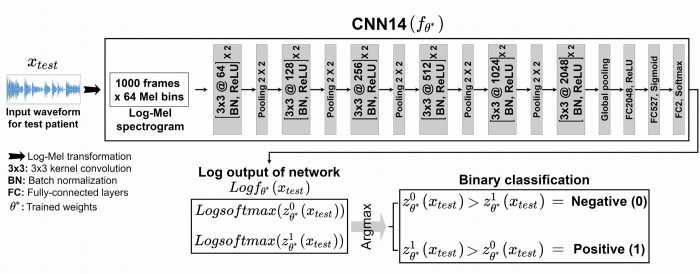
Configuration of CNN14. CNN14 is a representative network developed for audio-based classification, which converts input waveform into a log-Mel spectrogram (i.e., converts one-dimensional information into two-dimensional image type information) and makes the CNN appended to the spectrogram. The CNN is composed of convolution, batch normalization, and FC layers. In this study, the output size of the last layer was resized as two for binary classification, and transfer learning was adopted.

As a neural network model for audio tagging, we adopted a CNN named CNN14 [30] as our base model of AI, since it is suitable for using a log-Mel spectrogram as the model input. CNN14 is a model modified by Kong et al. [30] to be more suitable for audio tagging, based on the CNN structure called VGG [35] and consists of 14 layers. The detailed configuration of CNN14 is illustrated in Fig 4. CNN14 has a total of six convolutional layers, and each convolutional block is composed of two convolutional layers with a 3 × 3 kernel size. The number following the character @ indicates the number of feature maps. In this network, batch normalization (BN) [36] and Rectified Linear Unit (ReLU) nonlinearity [37] were applied between each convolutional layer, downsampling was performed using average pooling of 2 × 2 size before each convolutional block, and global average pooling [38] was applied after the last convolutional layer to extract the representative value of the two-dimensional feature map for each channel. As our task involved binary classification, we modified the original CNN14 to perform binary classification by adding a two-dimensional fully connected (FC) layer to the end of CNN14 (i.e., total of 15 layers). This modification makes the output dimension of the modified CNN14 become two so that it is possible to provide binary classification probability vectors for both classes. Each waveform (i.e., the pre-processed entire waveform file in the case of baseline model and the pre-processed waveform file of each individual patch divided into N pieces in the case of proposed model) is converted into a two-dimensional image of log-Mel spectrogram as shown in Fig 3. This spectrogram image is given as input to our CNN14, and the prediction results for binary classification are provided as a network output of a two-dimensional vector. This output vector is a softmax probability value that distinguishes whether the result is positive or negative by selecting the value of the larger index among the two values.

In all experiments, we set the mini-batch size to 32, set the epoch to 10,000, and used binary cross-entropy loss and the Adam optimizer with an initial learning rate of 0.001. Referring to existing AI-related studies in which transfer learning improved performance [39–41], we also adopted transfer learning [39] and set the parameters of CNN14 pre-trained on a large-scale AudioSet [42] dataset as initial parameters for our training. We set the sampling rate of the audio source to 32,000, window size to 1,024, hop size to 320, and window type to Hann as this approach has been regarded as suitable for audio signal processing [30]. We applied two data augmentation techniques, Mixup [43] and SpecAugment [44], to learn the network more effectively even with a limited number of training samples. Mixup creates new data by mixing two different sound sources and their labels into one, and SpecAugment augments data through masking in the frequency and time domains on a Mel spectrogram. For the experimental implementation environment, we used GPU GeForce GTX 1080Ti, CPU Intel® Xeon® CPU E5-2620 v4, and the Pytorch library.

### Evaluation metrics for measuring classification performance

We evaluated the classification performance according to the following five statistical analyses: area under the curve (AUC) for receiver operator characteristic (ROC), accuracy, precision, and confusion matrix.

Accuracy is denoted by the percentage of the total number of test samples that the network identified in the true labels. Precision denotes the class-wise averages of the proportions detected correctly among all samples detected by the target class.

Because the tasks were built as binary-label classifications, we expressed the hypokinetic dysarthria (H), ataxia (A), and none (N) cases as H, A, and N and calculated five true positive (TP), false positive (FP), and false negative cases by selecting a target label i∈{H, A} as positive and the other labels to exclude the label as negative (i.e., case 1 (H vs. others) and case 2 (A vs. others), respectively), selecting both labels H and A as positive and negative, respectively (i.e., case 3 (H vs. A)), selecting a target label i∈{H, A} as positive, and regarding the label N as negative (i.e., case 4 (H vs. N) and case 5 (A vs. N), respectively).

$$Accuracy=\frac{T_{p}+T_{n}}{D_{test}},$$ (11)

$$Precision=\frac{TP}{TP+FP}$$ (12)

where *T*_*i*_ is the number of testing samples with both labels and the estimate equal to i∈{*p* = 1, *n* = 0} = {positive, negative}, *D*_*test*_ is the total number of testing samples, *TP* and *FP* denote TP and FP, respectively, and *Precision* denotes the precision (i.e. positive (*p*) prediction value). All statistical analyses were performed by 5-fold cross-validation in the internal dataset [45]. We divided the entire dataset into five subsets. Thereafter, we trained the model using four subsets and evaluated it on the remaining single subset, thereby obtaining the five trained models individually. The five subsets of data for validation of each model were not duplicated, and the average performance through this 5-fold cross-validation was calculated to obtain the final AUC, accuracy, precision, and confusion matrix. Each fold of the autumn protocol consisted of 41 (H), 23 (A), and 15 (N) audio sources, and each fold of the number protocol consisted of 43 (H), 22 (A), and 16 (N) audio sources. Samples for each protocol were selected from different patients.

### Classification performance of the doctors

To compare the performance between AI and doctors (humans), three doctors in the third year of neurology residency went through the same 5-fold test for case 1 (hypokinetic dysarthria vs. others) and case 2 (ataxic vs. others). These doctors did not participate in the study data extraction and did not receive any clinical information about the recordings. Each doctor was evaluated separately and blinded from each other’s results. They were allowed to listen to the recordings only one time. To prevent the learning effect in the same case, they had to listen to 5-folds of the autumn protocol of case 1 followed by 5-folds of the autumn protocol of case 2 and then 5-folds of the number protocol of case 1 followed by the number protocol of case 2.

## Results

### Key performance evaluation of the proposed PWSI-AI compared to the baseline model

To demonstrate the superiority and usefulness of the proposed PWSI-AI, we compared it to the baseline model (i.e., the number of patches was set to one in PWSI-AI) in terms of the key performance measurement, AUC. We summarized the corresponding comparison results for each of the five cases in Table 3, where the macro-average AUCs were given through 5-fold cross-validations. As a result, in the case of the autumn protocol, we observed that the proposed PWSI-AI achieved an AUC performance improvement of 4% or more in all cases. In the case of the number protocol, we also demonstrated that the proposed PWSI-AI has an AUC performance improvement in all cases compared to the existing baseline AI model, of approximately 3% on average in all cases. Therefore, through these results, we proved the effectiveness of the proposed technology by demonstrating that the application of wave splitting and integration improves the classification performance of AI.

**Table 3:** Macro-average AUCs for the proposed PWSI-AI and baseline model.

Case/Protocol	Autumn	Number
Case 1 (H vs. O)	0.92 / 0.88 **(+0.04)**	0.92 / 0.87 **(+0.05)**
Case 2 (A vs. O)	0.93 / 0.86 **(+0.07)**	0.89 / 0.88 **(+0.01)**
Case 3 (H vs. A)	0.96 / 0.92 **(+0.04)**	0.95 / 0.93 **(+0.02)**
Case 4 (H vs. N)	0.86 / 0.80 **(+0.06)**	0.87 / 0.83 **(+0.04)**
Case 5 (A vs. N)	0.87 / 0.81 **(+0.06)**	0.87 / 0.84 **(+0.03)**

Data are presented as proposed/baseline (their difference). H, hypokinetic dysarthria; O, others; A, ataxic dysarthria; N, none

**Fig 5:**
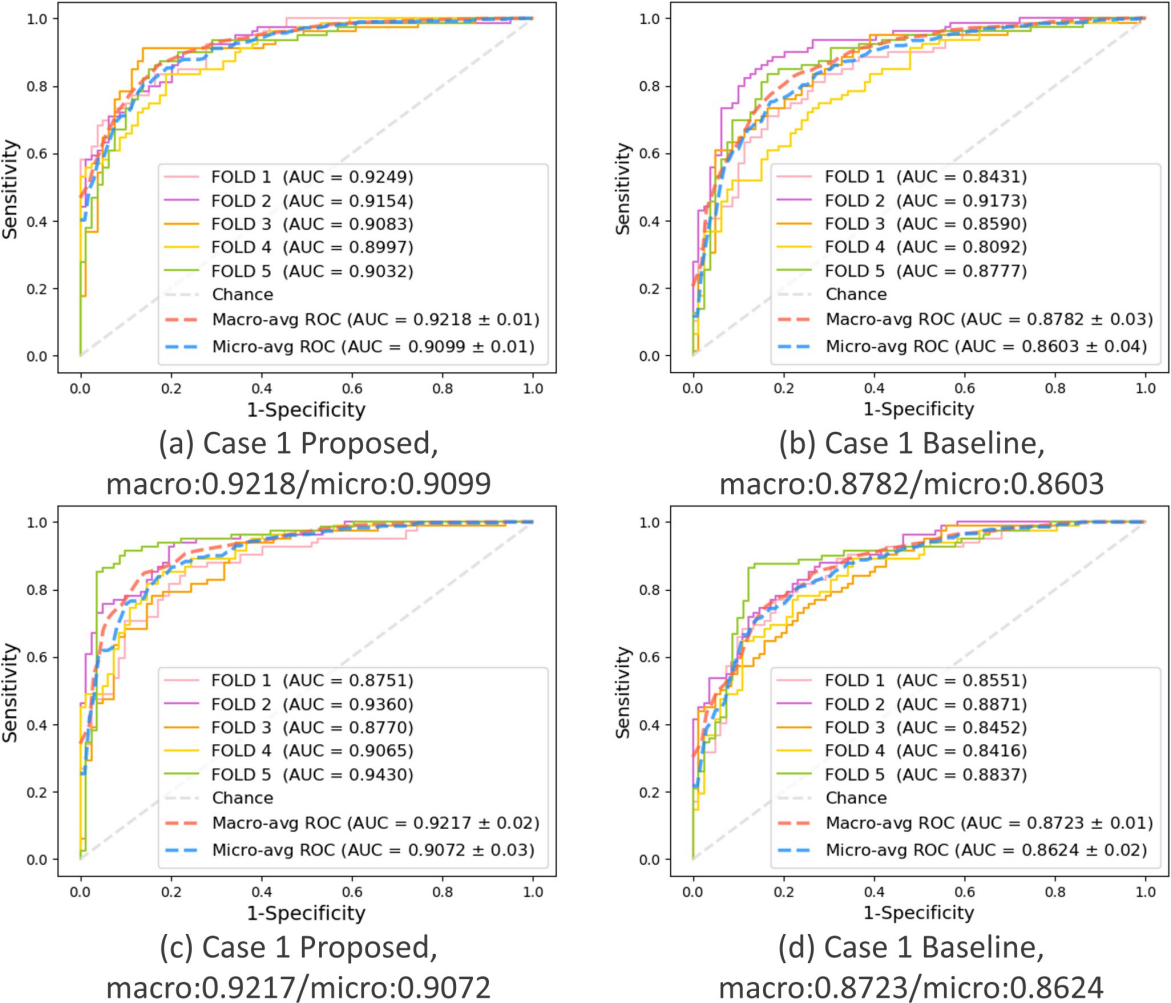
Case 1: Hypokinetic dysarthria vs. others. Comparison of the performance between the proposed and baseline schemes in terms of AUC. (a) Autumn + proposed, (b) Autumn + baseline, (c) Number + proposed, (d) Number + baseline.

**Fig 6:**
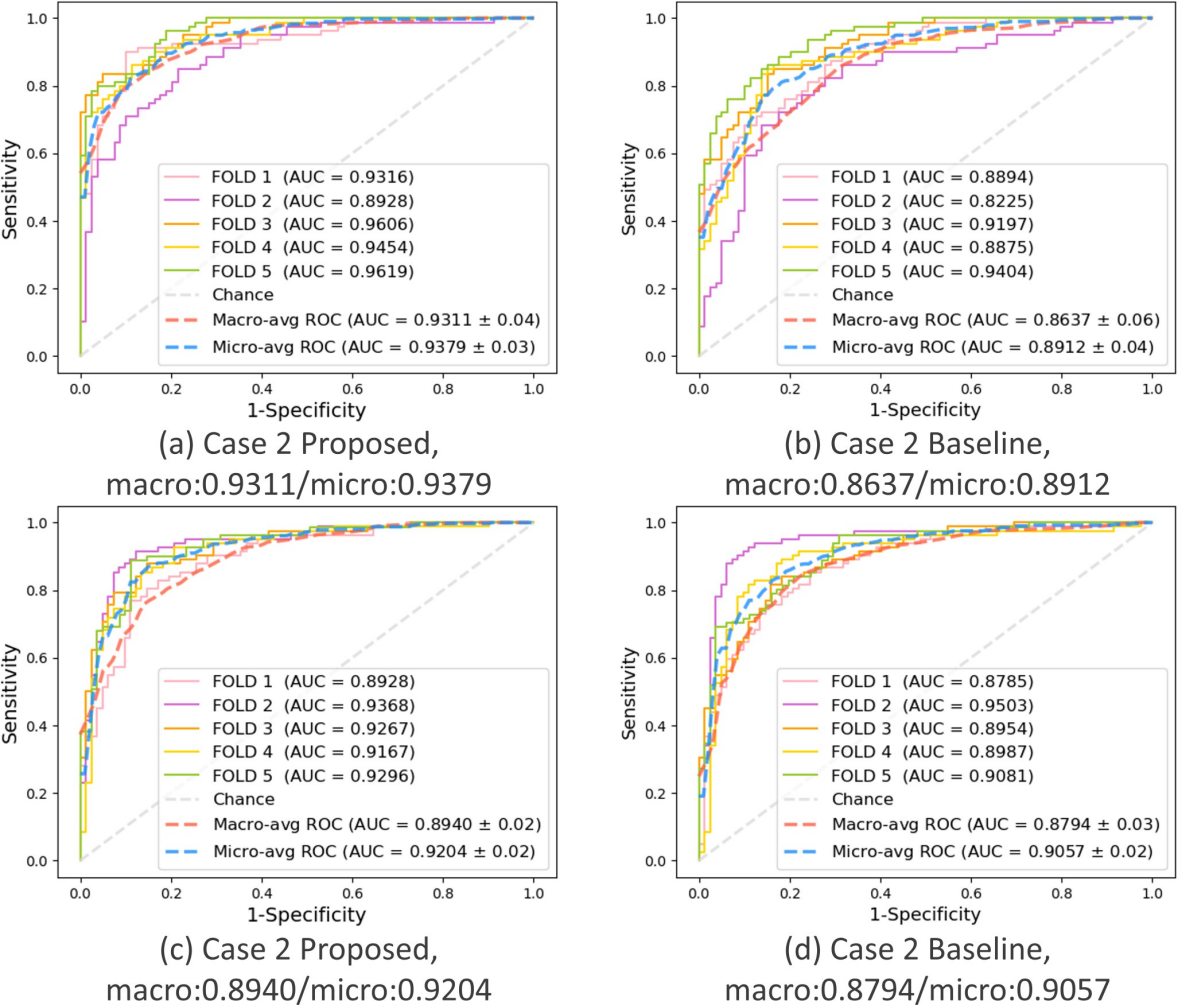
Case 2: Ataxia vs. others. Comparison of the performance between the proposed and baseline schemes in terms of AUC. (a) Autumn + proposed, (b) Autumn + baseline, (c) Number + proposed, (d) Number + baseline.

**Fig 7:**
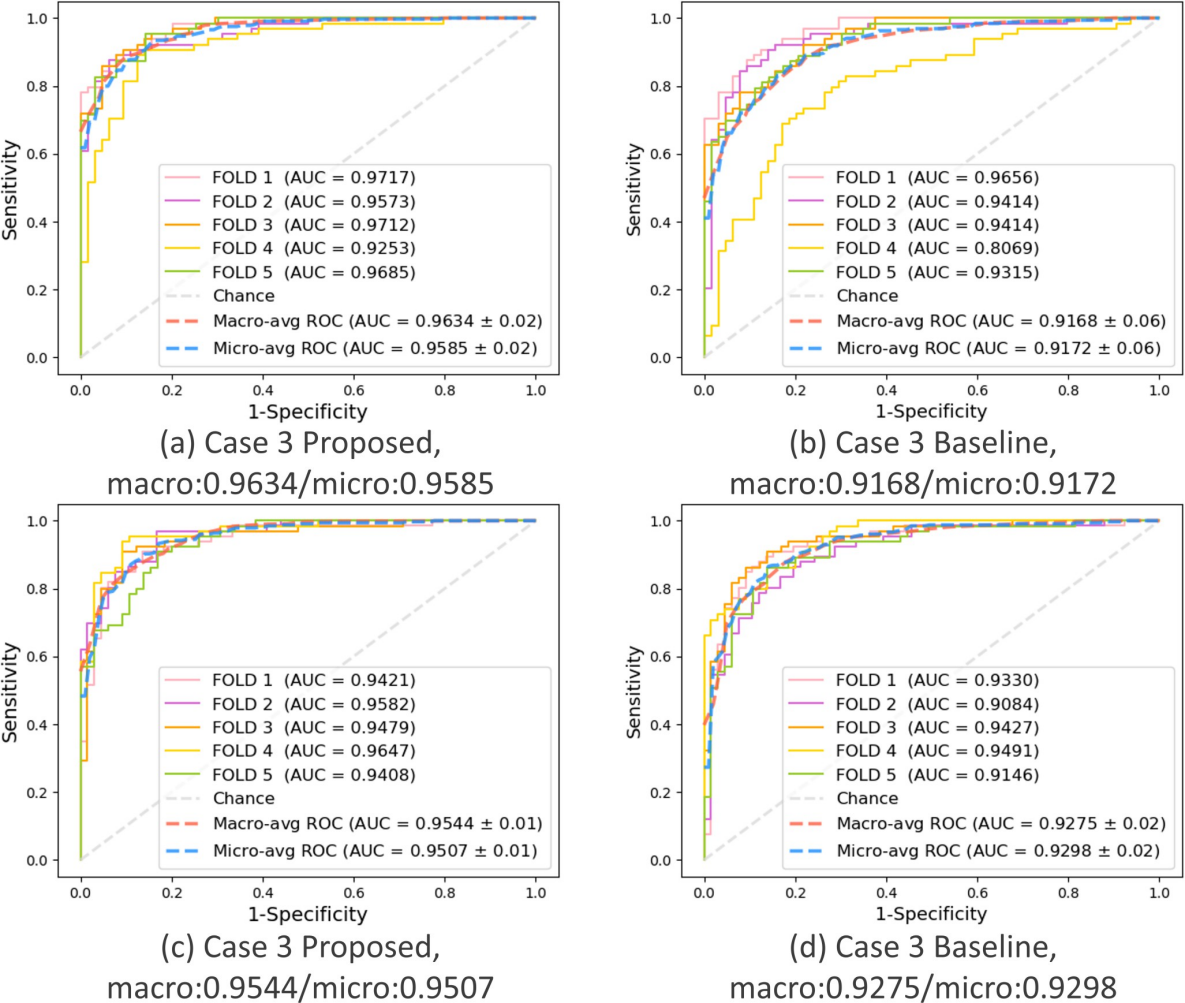
Case 3: Hypokinetic dysarthria vs. ataxia. Comparison of the performance between the proposed and baseline schemes in terms of AUC. (a) Autumn + proposed, (b) Autumn + baseline, (c) Number + proposed, (d) Number + baseline.

This key result is supported by the 5-fold individual ROC curves drawn for each case of the proposed PWSI-AI and the baseline model and the micro- and macro-average ROC curves, AUCs, and SDs based on the 5-fold results shown in Figs 5–7. These figures show that the proposed scheme also improved the micro-average AUCs of the baseline for cases 1, 2, and 3 (e.g., for case 3 in (a) and (b) of the autumn protocol in Fig 7, the proposed scheme (AUC: 0.9634) improved the micro-average AUC of the baseline (AUC: 0.9168) by more than 4%), thereby supporting the objectivity of the proposed technique and experiments. Specifically, no significant difference between the micro-average AUC and macro-average AUC for each model was observed, and our validation data were confirmed to be well-balanced for each class in each fold.

### Performance evaluation of the proposed PWSI-AI

Tables 4 and 5 can be used to examine the clinical effectiveness of the proposed technique and detail the AUCs, accuracy, precision, and convolution matrix for each of the 5-type classification cases. The average and SD were derived from each of the 5-fold cross-validations.

**Table 4:** AUCs, accuracy, and precision for the proposed PWSI-AI.

Modality		Autumn	Number
Case	Metrics	Validation results of AI	
**Case 1: Hypo Vs. Others**	Macro-avg AUC	0.9218 ± 0.01	0.9217 ± 0.02
	Micro-avg AUC	0.9099 ± 0.01	0.9072 ± 0.03
	Accuracy, %	81.77 ± 2.62	83.88 ± 4.13
	Precision, %	81.49 ± 2.81 (Hypo: 86.45 ± 3.38, Others: 76.53 ± 6.66)	83.8 ± 4.32 (Hypo: 85.68 ± 7.14, Others: 81.93 ± 11.11)
**Case 2: Ataxia Vs. Others**	Macro-avg AUC	0.9311± 0.04	0.8940 ± 0.02
	Micro-avg AUC	0.9379± 0.03	0.9204 ± 0.02
	Accuracy, %	84.05 ± 2.91	77.99 ± 4.8
	Precision, %	82.14 ± 5.7 (Ataxia: 77.71 ±13.54, Others: 86.57 ±3.64)	80.15 ± 3.17 (Ataxia: 84.78 ± 8.47, Others: 75.51 ± 8.43)
**Case 3: Hypo Vs. Ataxia**	Macro-avg AUC	0.9634 ± 0.02	0.9544 ± 0.01
	Micro-avg AUC	0.9585 ± 0.02	0.9507 ± 0.01
	Accuracy, %	89.04 ± 5.28	85.93 ± 2.74
	Precision, %	88.5 ± 5.71 (Hypo: 90.31 ± 4.28, Ataxia: 86.68 ± 7.42)	85.66 ± 2.46 (Hypo: 86.62 ± 6.67, Ataxia: 84.7 ± 8.2)
**Case 4: Hypo Vs. None**	Macro-avg AUC	0.8605 ± 0.03	0.8696 ± 0.05
	Micro-avg AUC	0.8814 ± 0.03	0.8907 ± 0.04
	Accuracy, %	75.61 ± 4.22	73.82 ± 4.22
	Precision, %	77.02 ± 2.64 (Hypo: 73.88 ± 6.84, None: 80.17 ± 5.01)	76.08 ± 7.1 (Hypo: 71.28 ± 7.16, None: 80.88 ± 17.7)
**Case 5: Ataxia Vs. None**	Macro-avg AUC	0.8657 ± 0.07	0.8652 ± 0.09
	Micro-avg AUC	0.8565 ± 0.07	0.8514 ± 0.09
	Accuracy, %	74.96 ± 6.34	76.67 ± 11.94
	Precision, %	73.64 ± 6.54 (Ataxia: 80.28 ± 7.78, None: 67 ± 9.75)	76.68 ± 10.95 (Ataxia: 76.52 ± 18.64, None: 76.84 ± 6.76)

Data are presented as the mean ± SD. AI, artificial intelligence; AUC, area under the curve, Hypo, hypokinetic dysarthria

**Table 5:** Confusion matrices for the proposed PWSI-AI.

Protocol: Autumn					Protocol: Number				
**Case 1: Hypo Vs. Others**	Confusion matrix		Predicted		**Case 1: Hypo Vs. Others**	Confusion matrix		Predicted	
			Hypo	Others				Hypo	Others
	Actual	Hypo	35.8 ±1.79	5.6 ±1.34		Actual	Hypo	37.0 ±2.92	6.2 ±3.11
		Others	8.8 ±2.39	28.8 ±2.86			Others	7.0 ±4.36	31.6 ±4.1
**Case 2: Ataxia Vs. Others**	Confusion matrix		Predicted		**Case 2: Ataxia Vs. Others**	Confusion matrix		Predicted	
			Ataxia	Others				Ataxia	Others
	Actual	Ataxia	17.4 ±3.05	5.0 ±3.08		Actual	Ataxia	18.8 ±1.64	3.4 ±1.95
		Others	7.6 ±2.07	49.0 ±2.12			Others	14.6 ±5.08	45.0 ±5.0
**Case 3: Hypo Vs. Ataxia**	Confusion matrix		Predicted		**Case 3: Hypo Vs. Ataxia**	Confusion matrix		Predicted	
			Hypo	Ataxia				Hypo	Ataxia
	Actual	Hypo	37.4 ±2.07	4.0 ±1.73		Actual	Hypo	37.4 ±2.61	5.8 ±2.95
		Ataxia	3.0 ±1.73	19.4 ±1.52			Ataxia	3.4 ±1.82	18.8 ±1.79
**Case 4: Hypo Vs. None**	Confusion matrix		Predicted		**Case 4: Hypo Vs. None**	Confusion matrix		Predicted	
			Hypo	None				Hypo	None
	Actual	Hypo	30.6 ±3.05	10.8 ±2.77		Actual	Hypo	30.8 ±3.19	12.4 ±3.05
		None	3.0 ±0.71	12.2 ±1.1			None	3.2 ±3.03	13.2 ±2.59
**Case 5: Ataxia Vs. None**	Confusion matrix		Predicted		**Case 5: Ataxia Vs. None**	Confusion matrix		Predicted	
			Ataxia	None				Ataxia	None
	Actual	Ataxia	18.0 ±2.0	4.4 ±1.67		Actual	Ataxia	17.0 ±4.18	5.2 ±4.09
		None	5.0 ±1.41	10.2 ±1.64			None	3.8 ±1.1	12.6 ±1.14

Data are presented as the mean ± SD. Hypo, hypokinetic dysarthria

Table 4 shows that in both cases 1 and 2, AI correctly detects hypokinetic dysarthria or ataxia in an environment with all three dysarthria types (i.e., hypokinetic dysarthria, ataxia, and none), with an AUC performance of more than 0.9 and 0.89 and accuracy and precision more than 80% and 77% in the autumn and number protocols, respectively. The autumn protocol showed higher performance than the number protocol because the voice length of the autumn protocol was longer than that of the number protocol. In the autumn protocol, we divided the waveform into ten but divided the value into three in the number protocol. The voice data for the number protocol were only tripled, but in the autumn protocol, the data were augmented ten times, improving the AI diagnosis performance. Notably, for both protocols, AI performed binary classification between hypokinetic dysarthria and ataxia with a high AUC (>0.95) and high accuracy (>85%), as shown in case 3 in Table 4. We also observed in cases 4 and 5 that AI performs the binary classification between hypokinetic dysarthria or ataxia and none, with an AUC of ≥0.85 and accuracy of about 75% (Table 4).

To confirm the results in Table 4, we also developed Table 5 to assess the confusion matrices. The results in Table 5 confirm that AI successfully performs classification (e.g., with high precision and accuracy of more than 73% in all cases, as shown in Table 4), even if there are some imbalances between the data of each class.

### Comparison of the performance between AI and doctors

**Fig 8:**
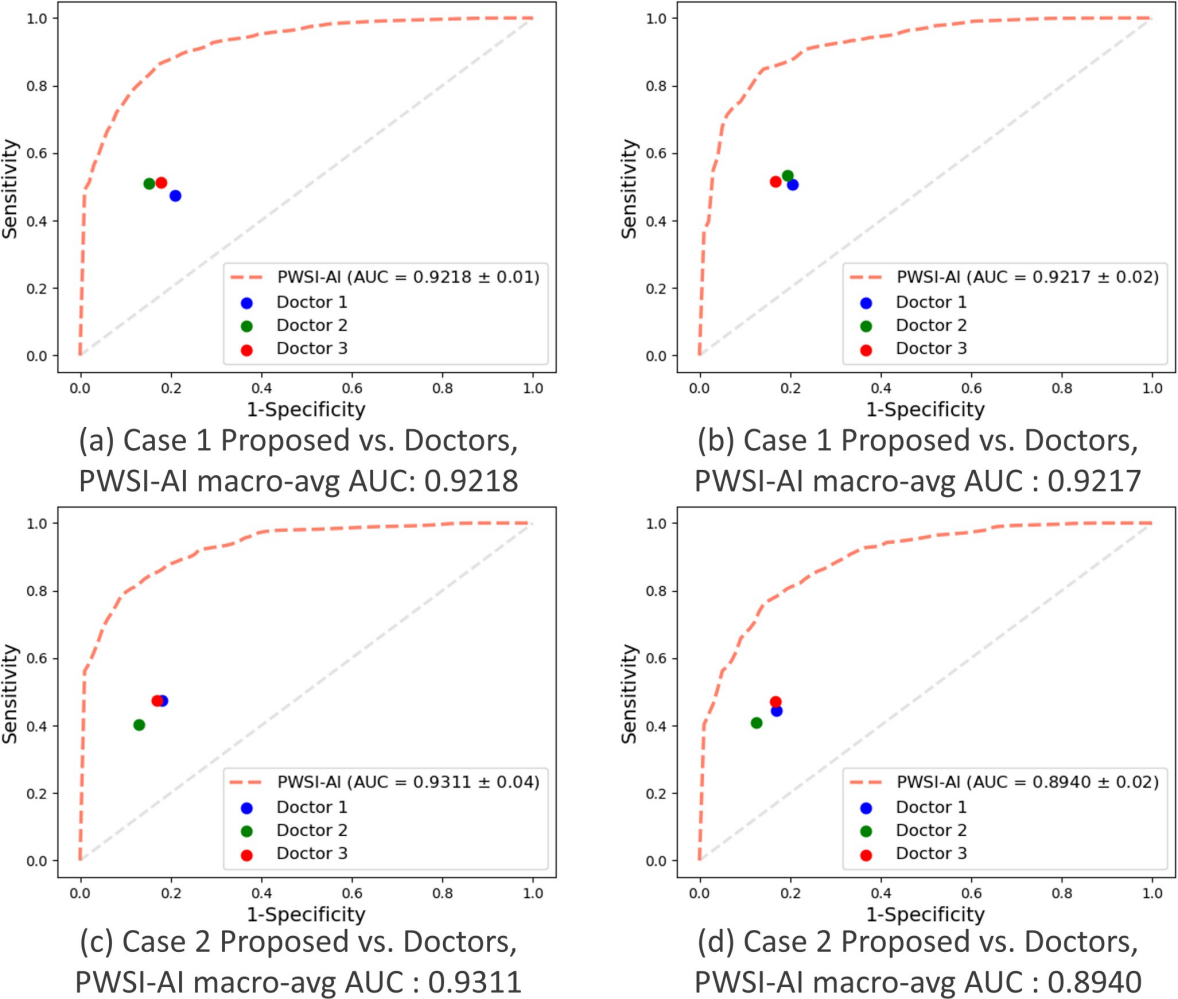
Comparison of the performance between the proposed PWSI-AI and doctors in terms of the AUC. (a) and (c) Autumn protocol, (b) and (d) Number protocol.

This section compares the results between the proposed PWSI-AI and neurology resident doctors in discriminating a specific dysarthria type. We provided the same 5-fold data of case 1 (hypokinetic dysarthria vs. others) and case 2 (ataxia vs. others), used to verify the proposed PWSI-AI, to three resident doctors. The detailed setup for this step has been introduced above, and the performance results are presented in Fig 8 (sensitivity, specificity) and Table 6 (accuracy, precision). Fig 8 shows that the ROC curve of the proposed AI technology was different by at least 0.1 in each direction of the x- and y-axes compared to the resident doctors. These results show that the proposed AI technology has more than 10% higher sensitivity and specificity than the resident doctors. Similarly, as shown in Table 6, the proposed AI technology has more than 5% higher accuracy and precision than resident doctors in all protocols in cases 1 and 2. These results prove the effectiveness of the proposed AI technology by suggesting that it can supplement the diagnosis of doctors who are not voice-based diagnostic specialists.

**Table 6:** Comparison between the proposed PWSI-AI and doctors.

	Doctor 1	Doctor 2	Doctor 3	Mean Doctors	PWSI-AI
**(C1/A) Accuracy**	47.4 ± 8.4	51.1 ± 9.4	51.2 ± 5.4	**49.9 ± 7.7**	**81.8 ± 2.6**
**(C1/A) Precision**	71.5 ± 11.9	77.3 ± 8.8	75.9 ± 10.4	**74.9 ± 10.4**	**81.5 ± 2.8**
**(C1/N) Accuracy**	50.8 ± 7.5	53.5 ± 3.0	51.7 ± 6.9	**52.0 ± 5.8**	**83.9 ± 4.1**
**(C1/N) Precision**	73.3 ± 9.4	74.5 ± 9.4	78.5 ± 9.9	**75.5 ± 9.54**	**83.8 ± 4.3**
**(C2/A) Accuracy**	47.4 ± 10.0	40.2 ± 15.7	47.4 ± 9.7	**45.0 ± 11.8**	**84.1 ± 2.9**
**(C2/A) Precision**	51.6 ± 11.4	51.3 ± 17.2	53.2 ± 13.0	**52.0 ± 13.9**	**82.1 ± 5.7**
**(C2/N) Accuracy**	44.6 ± 0.07	41.0 ± 17.8	47.2 ± 8.6	**44.3 ± 11.1**	**78.0 ± 4.8**
**(C2/N) Precision**	52.4 ± 10.7	54.9 ± 13.3	55.2 ± 11.8	**54.2 ± 11.9**	**80.2 ± 3.2**

C1 and C2 (A and N) indicate cases 1 and 2 (autumn and number protocols), respectively.

The values are given as the mean and SD.

## Discussion

Ataxic and hypokinetic dysarthria are important clinical clues for diagnosing and managing many neurodegenerative diseases, including cerebellar ataxia and parkinsonian diseases. This was the first study to develop automated analyses of speech recordings to differentiate ataxic and hypokinetic dysarthria. Our AI model, PWSI-AI-AC, showed reliable performance in differentiating ataxic and hypokinetic dysarthria, intrinsically augmenting data to effectively classify the types even with a small number of training samples. In all the tasks, the performance parameters of our AI model were significantly better than those of resident doctors.

As the elderly population and the prevalence of neurodegenerative diseases increase globally due to increased life expectancy, this automated program for the differential typing of dysarthria can facilitate the diagnosis and assessment of the severity or stage of diseases [1]. In particular, detecting hypokinetic dysarthria, which occurs in patients with PD, is needed for the proper management of PD of which prevalence is growing worldwide [8, 9]. Furthermore, ataxic dysarthria is a relatively common presentation of ataxia in pediatric and adult populations due to various etiologies and is also important to detect [10, 11]. Movement disorders such as atypical parkinsonism tend to present as either the hypokinetic or ataxic type or a mixture; this distinctive feature may make the differentiation efficient. Therefore, detecting hypokinetic or ataxic dysarthria can be a major clue in differentiating Parkinson plus syndromes.

Identifying the presence of ataxic or hypokinetic dysarthria in patients based on AI analysis will enable general doctors to provide more accurate differential diagnoses. As seen in the current study, it is difficult for general doctors to distinguish the types of dysarthria. It can even be challenging for diagnostic experts to differentiate PD from atypical parkinsonism based on neurological examination. The accuracy of clinical diagnosis for PD by non-specialists was reported to be 73.8% (95% CI 67.8%–79.6%), and the most frequent misdiagnosis in the clinical setting is atypical parkinsonism [46]. In the process of diagnosis in which doctors combine all available clinical clues, speech abnormality could play a significant role.

Older adults with neurodegenerative diseases often have limitations in ambulation, which can inhibit them from visiting the hospital in person on a regular basis. Therefore, assessing patients with dysarthria using AI remotely on a regular basis could help determine the progression or stage of the disease and develop a better management plan. More specifically, since dysarthria can be an important indirect sign of dysphagia, our AI can be used to screen for dysarthria, the risk of dysphagia in PD, and atypical parkinsonism. In addition, AI detection of hypokinetic dysarthria remotely can be utilized to detect dopaminergic response as the disease progresses [47, 48].

There was a significant and larger difference than expected in the performance of our AI model and the resident doctors. As we mentioned before, neurological diagnosis can be challenging for general doctors, even resident doctors based in a neurology department. Nevertheless, the test conditions could have influenced the performance of the resident doctors. They only had one chance to listen to the recordings. Furthermore, they had to listen to 800 recordings for the 5-folds test. Accordingly, there could have been a time limitation for listening to one recording, and doctors could have had a tendency to decide the type with less information. Moreover, they reported fatigue while listening to 80 recordings at once, which could have led to them making mistakes. Our study shows how AI can complement the limitations that humans have and produce better and more consistent performance than neurology resident doctors in the same task.

Our AI model was better at detecting ataxic or hypokinetic dysarthria from other diseases than cases without incidence, likely due to the relatively insufficient number of samples without incidence, compared to the cases of hypokinetic and ataxic dysarthria. Nevertheless, because AI can classify the hypokinetic dysarthria cases from the normal group with an AUC of about 0.90 and an accuracy of 75%, this finding can be interpreted as a meaningful result considering the difficulty for phonetically and clinically distinguishing between the hypokinetic dysarthria and normal groups [49].

Almost half of the cases of each type of dysarthria were mild in severity, and the majority were less than moderate, which shows the reliability of our model to differentiate even mild dysarthria. This data characteristic of our model shows the promising performance in early detection of neurodegenerative disease in which dysarthria is usually mild or minimal. Furthermore, although we developed the AI model using only the presence or absence of a type of dysarthria and not incorporating the severity, the fact that our AI model resulted in an ROC curve with high AUC, as shown in Figs 5–7, implies that it is clinically possible to quantitatively determine disease severity through the ROC curve with our AI technology. However, improvement sensitivity is needed with a larger number of cases with varying disease severity.

We developed a patch-based approach called PWSI-AI-AC, which intrinsically augmented the data ‘*N*’ times compared to the baseline. This approach helped improve the classification performance of dysarthria types with the same number of patients. Our method can be used in overcoming the limited numbers of data, a general limitation of previous studies. Using 409 recordings, we have achieved a detection accuracy similar to that obtained by Onur et al., who used 33,877 sound recordings to achieve an accuracy of 89.75% in detecting PD from healthy controls [19]. Previous studies performed data augmentation in the inter-sample [43] or frequency [44] domain, but this study performed data augmentation in the time domain (i.e., via the wave splitting), thus ensuring technical independence. In addition, while the existing data augmentation techniques are designed to be used only in the learning process and not in the inference process, the proposed data augmentation technique was applied (i.e., the wave splitting is performed) in both learning and inference processes, thereby ensuring the consistency and additional diversity gains (jointly considering multiple diagnosis results individually obtained by interval for one patient’s wavegram) in the inference process. Finally, the proposed technique has advanced diagnostic performance compared to the baseline via the integration that colligates the diagnostic results of augmented (i.e., patch-wise wave split) individual data for each patient.

In our experiments using PWSI-AI-AC (e.g., Table 4), the number protocol showed a similar performance to the autumn protocol. The length of the number protocol was at least three times shorter than that of the number protocol; this result suggests that the proposed PWSI-AI-AC provides sufficiently high diagnostic performance and is robust to the sound source length. Long protocols are conventionally preferred for precise diagnosis by doctors, but the short protocol can help shorten the examination time required. Therefore, the proposed PWSI-AI-AC is expected to satisfy a variety of clinical needs.

Our model analyzed sentence-based, contextual speech (e.g., autumn and number protocols) assessments, whereas most of the previous studies [50–54] analyzed continuing sound of the vowel “a” or diadochokinetic (DDK, e.g., “pa”-“ta”-“ka”) tasks. Contextual speech is essential in evaluating the integrated function of all aspects of speech, while the sound of “a” or DDK has limited value as the assessment of speed or regularity of articulatory movements [55]. Therefore, our model can be used to learn the integrated information of speech, not just part of the speech component. Quan et al. [54] conducted a comparative performance analysis between pronunciation- (i.e., “a”) and short sentence-based protocols with an AI-based diagnostic system and showed that the sentence-based protocol outperformed the pronunciation-based protocol even though both protocols captured 5 s in voice length. Our PWSI-AI-AC approach can also be more appropriate for sentenced-based data like contextual speech because it includes different syllables every second. In contrast, vowel or DDK tasks repeat the same syllables, resulting in little difference among the patch data (i.e., the proposed method makes a diagnosis by taking advantage of diversity through voice source multi-segmentation).

This study had several limitations. First, this was a single-center, single-ethnicity study. As a result, the amount of the data in each dysarthria group was not equalized, and the group without dysarthria was relatively small. However, we included pure hypokinetic and ataxic dysarthria with similar severity, improving the data quality. Even with our limited dataset, we could acquire a certain level of accuracy and developed an AI model, PWSI-AI-AC. Second, we did not enroll a normal matched elderly population as a control group to compare with the hypokinetic and ataxic dysarthria cases. However, this study focused on the differential diagnosis of dysarthria in patients and not just on screening healthy subjects. Third, we used binary classification (hypokinetic and ataxic dysarthria) but did not examine the incidence of other dysarthria such as spastic, flaccid, or mixed types. Future research should assess whether our AI model can detect the predominant type among various dysarthria.

## Conclusions

The proposed patch-based AI diagnosis approach could intrinsically augment data to effectively classify dysarthria types even with a small number of training samples, demonstrating additional performance improvement compared to the existing AI models. Our findings demonstrate the potential usefulness of our model to collect sufficient data in clinically difficult environments. We found that ataxic and hypokinetic dysarthria could be detected and differentiated by our proposed AI with higher performance than neurology resident doctors. Therefore, this AI model could be used by physicians to screen for neurodegenerative diseases and assist experts with the differential diagnosis of neurodegenerative diseases. Our model can be integrated with other AI models to facilitate highly accurate differential diagnoses of neurodegenerative disorders. AI models that differentiate brain magnetic resonance or PET imaging currently lack clinical information, and our model can address this significant gap. Further development should be undertaken to enable multi-class differentiation among various types of dysarthria such as spastic, flaccid, and even mixed cases using our AI model.

## Supporting information

S1 File Example for number protocol. (WAV)

S2 File Example for autumn protocol. (WAV)

## References

[1] Yorkston KM. The Degenerative Dysarthrias: A Window into Critical Clinical and Research Issues. Folia Phoniatrica et Logopaedica. 2007;59(3):107–17. doi: 10.1159/000101769 17556854

[2] Finch E, Rumbach AF, Park S. Speech pathology management of non-progressive dysarthria: a systematic review of the literature. Disability and Rehabilitation. 2020;42(3):296–306. doi: 10.1080/09638288.2018.1497714 30286661

[3] Tjaden K. Speech and Swallowing in Parkinson’s Disease. Top Geriatr Rehabil. 2008;24(2):115–26. doi: 10.1097/01.TGR.0000318899.87690.44 . 19946386 PMC2784698

[4] Kluin KJ, Gilman S, Lohman M, Junck L. Characteristics of the dysarthria of multiple system atrophy. Arch Neurol. 1996;53(6):545–8. Epub 1996/06/01. doi: 10.1001/archneur.1996.00550060089021 . 8660157

[5] Sidtis JJ, Ahn JS, Gomez C, Sidtis D. Speech characteristics associated with three genotypes of ataxia. J Commun Disord. 2011;44(4):478–92. Epub 2011/04/15. doi: 10.1016/j.jcomdis.2011.03.002 . 21592489 PMC3159076

[6] Rusz J, Bonnet C, Klempíř J, Tykalová T, Baborová E, Novotný M, et al. Speech disorders reflect differing pathophysiology in Parkinson’s disease, progressive supranuclear palsy and multiple system atrophy. J Neurol. 2015;262(4):992–1001. Epub 2015/02/17. doi: 10.1007/s00415-015-7671-1 . 25683763

[7] Kluin KJ, Gilman S, Foster NL, Sima AAF, D’Amato CJ, Bruch LA, et al. Neuropathological Correlates of Dysarthria in Progressive Supranuclear Palsy. Archives of Neurology. 2001;58(2):265–9. doi: 10.1001/archneur.58.2.265 11176965

[8] Ascherio A, Schwarzschild MA. The epidemiology of Parkinson’s disease: risk factors and prevention. The Lancet Neurology. 2016;15(12):1257–72. doi: 10.1016/S1474-4422(16)30230-7 27751556

[9] Ponjoan A, Garre-Olmo J, Blanch J, Fages E, Alves-Cabratosa L, Martí-Lluch R, et al. Epidemiology of dementia: prevalence and incidence estimates using validated electronic health records from primary care. Clin Epidemiol. 2019;11:217–28. doi: 10.2147/CLEP.S186590 . 30881138 PMC6407519

[10] Musselman KE, Stoyanov CT, Marasigan R, Jenkins ME, Konczak J, Morton SM, et al. Prevalence of ataxia in children: a systematic review. Neurology. 2014;82(1):80–9. Epub 2013/11/29. doi: 10.1212/01.wnl.0000438224.25600.6c ; PubMed Central PMCID: PMC3873624. 24285620 PMC3873624

[11] Pandolfo M, Manto M. Cerebellar and afferent ataxias. Continuum (Minneap Minn). 2013;19(5 Movement Disorders):1312–43. Epub 2013/10/05. doi: 10.1212/01.CON.0000436158.39285.22 . 24092292

[12] Kashyap B, Horne M, Pathirana PN, Power L, Szmulewicz D. Automated Topographic Prominence based quantitative assessment of speech timing in Cerebellar Ataxia. Biomedical Signal Processing and Control. 2020;57:101759.

[13] Mei J, Desrosiers C, Frasnelli J. Machine Learning for the Diagnosis of Parkinson’s Disease: A Review of Literature. Frontiers in Aging Neuroscience. 2021;13(184). doi: 10.3389/fnagi.2021.633752 34025389 PMC8134676

[14] Gupta S, Patil AT, Purohit M, Parmar M, Patel M, Patil HA, et al. Residual Neural Network precisely quantifies dysarthria severity-level based on short-duration speech segments. Neural Networks. 2021;139:105–17. doi: 10.1016/j.neunet.2021.02.008 33684609

[15] Hauptman Y, Aloni-Lavi R, Lapidot I, Gurevich T, Manor Y, Naor S, et al., editors. Identifying Distinctive Acoustic and Spectral Features in Parkinson’s Disease. Interspeech; 2019.

[16] Wu K, Zhang D, Lu G, Guo Z. Learning acoustic features to detect Parkinson’s disease. Neurocomputing. 2018;318:102–8.

[17] Perez M, Jin W, Le D, Carlozzi N, Dayalu P, Roberts A, et al. Classification of Huntington Disease using Acoustic and Lexical Features. Interspeech. 2018;2018:1898–902. Epub 2018/01/01. doi: 10.21437/interspeech.2018-2029 ; PubMed Central PMCID: PMC7685291. 33241056 PMC7685291

[18] Lauraitis A, Maskeliūnas R, Damaševičius R, Krilavičius T. Detection of Speech Impairments Using Cepstrum, Auditory Spectrogram and Wavelet Time Scattering Domain Features. IEEE Access. 2020;8:96162–72. doi: 10.1109/ACCESS.2020.2995737

[19] Karaman O, Çakın H, Alhudhaif A, Polat K. Robust automated Parkinson disease detection based on voice signals with transfer learning. Expert Systems with Applications. 2021;178:115013.

[20] Abayomi-Alli OO, Damaševičius R, Maskeliūnas R, Abayomi-Alli A, editors. BiLSTM with Data Augmentation using Interpolation Methods to Improve Early Detection of Parkinson Disease. 2020 15th Conference on Computer Science and Information Systems (FedCSIS); 2020 6–9 Sept. 2020.

[21] Jiao Y, Tu M, Berisha V, Liss J, editors. Simulating Dysarthric Speech for Training Data Augmentation in Clinical Speech Applications. 2018 IEEE International Conference on Acoustics, Speech and Signal Processing (ICASSP); 2018 15–20 April 2018.

[22] Kaya M, Karakuş S, Tuncer SA. Detection of ataxia with hybrid convolutional neural network using static plantar pressure distribution model in patients with multiple sclerosis. Computer Methods and Programs in Biomedicine. 2022;214:106525. doi: 10.1016/j.cmpb.2021.106525 34852958

[23] Wang YY, Gao K, Kloepper AM, Zhao Y, Kuruvilla-Dugdale M, Lever TE, et al., editors. DeepDDK: A Deep Learning based Oral-Diadochokinesis Analysis Software. 2019 IEEE EMBS International Conference on Biomedical & Health Informatics (BHI); 2019 19–22 May 2019. 10.1109/bhi.2019.8834506 PMC7451101 32864624

[24] Hughes AJ, Daniel SE, Kilford L, Lees AJ. Accuracy of clinical diagnosis of idiopathic Parkinson’s disease: a clinico-pathological study of 100 cases. J Neurol Neurosurg Psychiatry. 1992;55(3):181–4. Epub 1992/03/01. doi: 10.1136/jnnp.55.3.181 ; PubMed Central PMCID: PMC1014720. 1564476 PMC1014720

[25] Gilman S, Wenning GK, Low PA, Brooks DJ, Mathias CJ, Trojanowski JQ, et al. Second consensus statement on the diagnosis of multiple system atrophy. Neurology. 2008;71(9):670–6. doi: 10.1212/01.wnl.0000324625.00404.15 . 18725592 PMC2676993

[26] Höglinger GU, Respondek G, Stamelou M, Kurz C, Josephs KA, Lang AE, et al. Clinical diagnosis of progressive supranuclear palsy: The movement disorder society criteria. Movement disorders: official journal of the Movement Disorder Society. 2017;32(6):853–64. Epub 2017/05/03. doi: 10.1002/mds.26987 . 28467028 PMC5516529

[27] Abele M, Minnerop M, Urbach H, Specht K, Klockgether T. Sporadic adult onset ataxia of unknown etiology. J Neurol. 2007;254(10):1384. doi: 10.1007/s00415-007-0556-1 17934884

[28] Yoshida K, Kuwabara S, Nakamura K, Abe R, Matsushima A, Beppu M, et al. Idiopathic cerebellar ataxia (IDCA): Diagnostic criteria and clinical analyses of 63 Japanese patients. Journal of the Neurological Sciences. 2018;384:30–5. doi: 10.1016/j.jns.2017.11.008 29249373

[29] Darley FL, Aronson AE, Brown JR. Differential Diagnostic Patterns of Dysarthria. Journal of Speech and Hearing Research. 1969;12(2):246–69. doi: 10.1044/jshr.1202.246 5808852

[30] Kong Q, Cao Y, Iqbal T, Wang Y, Wang W, Plumbley MD. Panns: Large-scale pretrained audio neural networks for audio pattern recognition. IEEE/ACM Transactions on Audio, Speech, and Language Processing. 2020;28:2880–94.

[31] McFee B, Raffel C, Liang D, Ellis DP, McVicar M, Battenberg E, et al., editors. librosa: Audio and music signal analysis in python. Proceedings of the 14th python in science conference; 2015: Citeseer.

[32] Choi K, Fazekas G, Sandler M. Automatic tagging using deep convolutional neural networks. arXiv preprint arXiv:160600298. 2016.

[33] Kong Q, Yu C, Xu Y, Iqbal T, Wang W, Plumbley MD. Weakly labelled audioset tagging with attention neural networks. IEEE/ACM Transactions on Audio, Speech, and Language Processing. 2019;27(11):1791–802.

[34] Portnoff M. Time-frequency representation of digital signals and systems based on short-time Fourier analysis. IEEE Transactions on Acoustics, Speech, and Signal Processing. 1980;28(1):55–69.

[35] Simonyan K, Zisserman A. Very deep convolutional networks for large-scale image recognition. arXiv preprint arXiv:14091556. 2014.

[36] Ioffe S, Szegedy C, editors. Batch normalization: Accelerating deep network training by reducing internal covariate shift. International conference on machine learning; 2015: PMLR.

[37] Nair V, Hinton GE, editors. Rectified linear units improve restricted boltzmann machines. Icml; 2010.

[38] Lin M, Chen Q, Yan S. Network in network. arXiv preprint arXiv:13124400. 2013.

[39] Pan SJ, Yang Q. A survey on transfer learning. IEEE Transactions on knowledge and data engineering. 2009;22(10):1345–59.

[40] Torrey L, Shavlik J. Transfer learning. Handbook of research on machine learning applications and trends: algorithms, methods, and techniques. IGI global. 2010:242–64.

[41] Weiss K, Khoshgoftaar TM, Wang D. A survey of transfer learning. Journal of Big data. 2016;3(1):1–40.

[42] Gemmeke JF, Ellis DP, Freedman D, Jansen A, Lawrence W, Moore RC, et al., editors. Audio set: An ontology and human-labeled dataset for audio events. 2017 IEEE international conference on acoustics, speech and signal processing (ICASSP); 2017: IEEE.

[43] Zhang H, Cisse M, Dauphin YN, Lopez-Paz D. mixup: Beyond empirical risk minimization. arXiv preprint arXiv:171009412. 2017.

[44] Park DS, Chan W, Zhang Y, Chiu C-C, Zoph B, Cubuk ED, et al. Specaugment: A simple data augmentation method for automatic speech recognition. arXiv preprint arXiv:190408779. 2019.

[45] Zhang H, Wang A, Li D, Xu W, editors. DeepVoice: a voiceprint-based mobile health framework for Parkinson’s disease identification. 2018 IEEE EMBS International Conference on Biomedical & Health Informatics (BHI); 2018: IEEE.

[46] Rizzo G, Copetti M, Arcuti S, Martino D, Fontana A, Logroscino G. Accuracy of clinical diagnosis of Parkinson disease. Neurology. 2016;86(6):566. doi: 10.1212/WNL.0000000000002350 26764028

[47] Skodda S, Visser W, Schlegel U. Short- and long-term dopaminergic effects on dysarthria in early Parkinson’s disease. Journal of Neural Transmission. 2010;117(2):197–205. doi: 10.1007/s00702-009-0351-5 20012657

[48] Davidson MB, McGhee DJ, Counsell CE. Comparison of patient rated treatment response with measured improvement in Parkinson’s disease. J Neurol Neurosurg Psychiatry. 2012;83(10):1001–5. Epub 2012/05/26. doi: 10.1136/jnnp-2012-302741 . 22626942

[49] Wang BJ, Carter FL, Altman KW. Relationship between Dysarthria and Oral-Oropharyngeal Dysphagia: The present evidence. Ear, Nose & Throat Journal. 2020:0145561320951647. doi: 10.1177/0145561320951647 33044841

[50] Louzada T, Beraldinelle R, Berretin-Felix G, Brasolotto AG. Oral and vocal fold diadochokinesis in dysphonic women. Journal of Applied Oral Science. 2011;19:567–72. doi: 10.1590/s1678-77572011000600005 22230989 PMC3973456

[51] Wroge TJ, Özkanca Y, Demiroglu C, Si D, Atkins DC, Ghomi RH, editors. Parkinson’s disease diagnosis using machine learning and voice. 2018 IEEE Signal Processing in Medicine and Biology Symposium (SPMB); 2018: IEEE.

[52] Almeida JS, Rebouças Filho PP, Carneiro T, Wei W, Damaševičius R, Maskeliūnas R, et al. Detecting Parkinson’s disease with sustained phonation and speech signals using machine learning techniques. Pattern Recognition Letters. 2019;125:55–62.

[53] Palacios-Alonso D, Meléndez-Morales G, López-Arribas A, Lázaro-Carrascosa C, Gómez-Rodellar A, Gómez-Vilda P. MonParLoc: a speech-based system for Parkinson’s disease analysis and monitoring. IEEE Access. 2020;8:188243–55.

[54] Quan C, Ren K, Luo Z. A deep learning based method for Parkinson’s disease detection using dynamic features of speech. IEEE Access. 2021;9:10239–52.

[55] Flanagan JL. Speech analysis synthesis and perception: Springer Science & Business Media; 2013.

